# A Genome-Wide Association Study on Chronic HBV Infection and Its Clinical Progression in Male Han-Taiwanese

**DOI:** 10.1371/journal.pone.0099724

**Published:** 2014-06-18

**Authors:** Su-Wei Chang, Cathy Shen-Jang Fann, Wen-Hui Su, Yu Chen Wang, Chia Chan Weng, Chia-Jung Yu, Chia-Lin Hsu, Ai-Ru Hsieh, Rong-Nan Chien, Chia-Ming Chu, Dar-In Tai

**Affiliations:** 1 Clinical Informatics and Medical Statistics Research Center, Chang Gung University College of Medicine, Taoyuan, Taiwan; 2 Institute of Biomedical Sciences, Academia Sinica, Taipei, Taiwan; 3 Department of Biomedical Sciences, Graduate Institute of Biomedical Sciences, Chang Gung Molecular Medicine Research Center, Chang Gung University, Taoyuan, Taiwan; 4 Division of Hepatology, Department of Gastroenterology and Hepatology, Chang Gung Memorial Hospital, Linkou, Taiwan; 5 Department of Cell and Molecular Biology, College of Medicine and Molecular Medicine Research Center, Chang Gung University, Tao-Yuan, Taiwan; 6 Graduate Institute of Biostatistics, China Medical University, Taichung, Taiwan; University of Pisa, Italy

## Abstract

It is common to observe the clustering of chronic hepatitis B surface antigen (HBsAg) carriers in families. Intra-familial transmission of hepatitis B virus (HBV) could be the reason for the familial clustering of HBsAg carriers. Additionally, genetic and gender factors have been reported to be involved. We conducted a three-stage genome-wide association study to identify genetic factors associated with chronic HBV susceptibility. A total of 1,065 male controls and 1,623 male HBsAg carriers were included. The whole-genome genotyping was done on Illumina HumanHap550 beadchips in 304 healthy controls and HumanHap610 beadchips in 321 cases. We found that rs9277535 (HLA-DPB1, P = 4.87×10^−14^), rs9276370 (HLA-DQA2, P = 1.9×10^−12^), rs7756516 and rs7453920 (HLA-DQB2, P = 1.48×10^−11^ and P = 6.66×10^−15^ respectively) were significantly associated with persistent HBV infection. A novel SNP rs9366816 near HLA-DPA3 also showed significant association (P = 2.58×10^−10^). The “T-T-G-G-T” haplotype of the five SNPs further signified their association with the disease (P = 1.48×10^−12^; OR = 1.49). The “T-T” haplotype composed of rs7756516 and rs9276370 was more prevalent in severe disease subgroups and associated with non-sustained therapeutic response (P = 0.0262). The “G-C” haplotype was associated with sustained therapeutic response (P = 0.0132; OR = 2.49). We confirmed that HLA-DPB1, HLA-DQA2 and HLA-DQB2 loci were associated with persistent HBV infection in male Taiwan Han-Chinese. In addition, the HLA-DQA2 and -DQB2 complex was associated with clinical progression and therapeutic response.

## Introduction

It is common to observe clustering of chronic hepatitis B surface antigen (HBsAg) carriers and hepatocellular carcinoma (HCC) in families of affected individuals [1]–[5]. A considerable number of patients exposed to HBV in the early stage of life became chronic HBsAg carriers [3], [6]–[8]. Most of the children born to the mothers with active HBV replications became HBsAg carriers. This type of perinatal infection gives little room for genetic roles to participate [3]. However, horizontal transmission is as important as perinatal infection in HCC families. The total number of HBsAg carriers was higher in children of male HCC patients (high horizontal transmission) than in children of female HCC patients (high perinatal infection) [5]. Genetic roles could be operating in patients with the horizontal transmission as well as in those mothers with inactive HBV replications. Previous candidate gene studies suggested that human leukocyte antigen (HLA), cytokines, DNA repair and others were involved in HBV clearance or progression to hepatocarcinogenesis [8]–[10]. Recent genome-wide association studies (GWAS) confirmed that human gene variations in HLA-DP and HLA-DQ areas were associated with HBsAg persistence [11]–[12]. However, the clinical significance of these genes on HBV-related disease progression remained unclear. It could be shielded by multi-factorial problems associated with chronic HBV infection. It is well-known that the prevalence of HBsAg carriers in males is much higher than that in females [6]. Therefore, we conducted a GWAS in male Taiwan-Han Chinese to examine the differences between non-HBsAg carriers, inactive HBsAg carriers and active HBV related liver diseases.

## Materials and Methods

### Study Participants

A total of 1623 male case samples (321 in the GWAS scan, 646 in the first replication and 656 in the second replication) were recruited from Chang Gung Memorial Hospital (CGMH), Linkou or Taiwan Liver Cancer Network (TLCN). The cases with age>30 years were enrolled when they were seropositive for the HBsAg for >6 months and seronegative for the anti-hepatitis C antibody (anti-HCV). All of the patients denied HIV infection. The 1623 HBsAg carriers were classified into three groups. The first group includes unrelated HBsAg carriers with persistent normal alanine aminotransferase (ALT) level (PNALT) for >5 years and with HBV DNA<10^5^ cps/mL, which were carefully selected from HBsAg carriers who visited the carrier clinic of Chang Gung Memorial Hospital and were followed for a long run [13], [14]. The second group was comprised of unrelated chronic hepatitis B (CHB) patients with fluctuated ALT levels and HBV DNA>10^5^ cps/mL. The third group included unrelated HCC patients. The age and sample size information for this three-stage study is given in Table 1.

**Table 1:** Descriptive characteristic of the three-stage study samples in different clinical groups.

Characteristics	GWAS Scan		1^st^ replication		2^nd^ replication		Combined	
	Case (N = 321)	Control (N = 304)	Case (N = 646)	Control (N = 345)	Case (N = 656)	Control (N = 416)	Case (N = 1623)	Control (N = 1065)
Age (years), ***Mean (Std)***								
	51.02 (7.69)	48.78 (19.16)	49.64 (11.83)	52.26 (13.00)	50.56 (11.44)	55.04 (10.29)	50.29 (10.97)	52.35 (14.39)
Clinical History*, ***N (%)***								
PNALT group	69 (21.50)		187 (28.95)		212 (32.32)		468 (28.84)	
Age (years), ***Mean (Std)***								
	49.91 (7.27)		47.63 (11.11)		49.00 (10.93)		48.59 (10.53)	
CHB group	173 (53.89)		313 (48.45)		308 (46.95)		794 (48.92)	
Age (years), ***Mean (Std)***								
	50.73 (7.97)		48.08 (11.25)		48.93 (10.73)		48.99 (10.45)	
HCC group	79 (24.61)		146 (22.60)		136 (20.73)		361 (22.24)	
Age (years), ***Mean (Std)***								
	52.63 (7.24)		55.53 (12.09)		56.69 (11.75)		55.34 (11.15)	

The 304 male control samples for the first stage GWAS scan were obtained from the Taiwan Han Chinese Cell and Genome Bank (who had no liver related disease and were seronegative for HBsAg and anti-HCV) [15]. The 761 male control individuals in the two replication studies were selected from local residents of Taoyuan County, Taiwan through a project designated “Integrated Delivery System of Health Screening, Taoyuan, Taiwan”. We included Han-Chinese origin subjects with age>30 years. The inclusion criteria for the controls were seronegative for HBsAg and anti-HCV, and with normal liver biochemistry records. We did not use anti-Hepatitis B core protein (anti-HBc) in our inclusion criteria because nearly 90% of general population age>30 years were infected with HBV [16], [17]. Those anti-HBc negative subjects who have been living in Taiwan, an HBV endemic area, for more than 30 years were not excluded. They had chances to expose to HBV, but may be genetically resistant to HBV infection [3].

This study as well as the inform consent were approved by the Ethics Committee of Chang Gung Memorial Hospital (IRB 94-581). A written informed consent was obtained from all the participants before the study. This clinical investigation had been conducted according to the principles expressed in the Declaration of Helsinki.

### Genome-wide SNP Genotyping and Quality Control

The genomic DNA was extracted from peripheral blood lymphocytes using the MagNA Pure LC DNA Isolation Kit with automatic DNA isolation instruments (MagNA Pure LC II; Roche Diagnostics GmbH, Mannheim, Germany). In the first-stage GWAS scan, the genome-wide SNP genotyping was done by Genizon Biosciences (Quebec, Canada) using Illumina HumanHap610 beadchips in the case group; while the genotyping for the control group was done by DeCode Genetics (Reykjavik, Iceland) using Illumina HumanHap550 beadchips. Only the overlapping SNPs on both chips were included in analysis. For filtering high-quality genotype data and for enlarging the number of potentially associated SNPs, we carried out the following quality control procedures other than the widely-used standard of the call rate of 95% and a minor allele frequency (MAF) of 5%; we included SNPs if: (1) a successful call rate in both cases and controls was >90%; (2) an MAF in the controls was >1%; (3) the Hardy-Weinberg equilibrium (HWE) was not violated in the controls (HWE test P-value>10^−7^).

#### SNP genotyping

In the two replication studies, the genotyping of the SNPs identified by GWAS was performed using TaqMan Genotyping assays (Applied Biosystems Inc.) or SequenomMassARRAY System (San Diego, CA). The experiment with TaqMan assays was done by Vita Genomics (New Taipei City, Taiwan), and the experiment with SequenomMassArray was done by the Academia Sinica National Genotyping Center (Taipei, Taiwan).

### Statistical Analysis

We performed three single-locus association tests: genotype test, allele test and Cochran-Armitage trend test to compare allele and genotype frequencies assuming three modes of inheritance: additive, dominant and recessive between the cases and the controls for each SNP. To increase the chances of identifying potentially associated SNPs in the GWAS scan, a SNP marker with –log_10_(P-value) ≥5 in any of the three above mentioned tests would be considered for further analysis. In the first and the second replication studies, the significance level after Bonferroni correction for multiple comparisons (P = 0.05 divided by the number of SNPs in the analysis) was used instead. The meta-analysis was then carried out using the Mantel-Haenszel method. The odds ratios (ORs) and 95% confidence intervals (CIs) were calculated by logistic regression analysis with age adjustment. The linkage disequilibrium (LD) map and values for the analyzed SNPs were generated by the Haploview software [18]. Haplotypes were inferred and maximum likelihood estimates of haplotype frequencies were generated by using the expectation-maximization (EM) algorithm. Those haplotype categories with estimated frequencies of less than 5% were combined into one category called “All other”. Effects of each haplotype category with estimated frequencies of at least 5% on persistent HBV infection were then assessed between the HBsAg carriers and the controls using logistic regression.

To detect the effect of potential population stratification, we used the principle component analysis (PCA) based software “EIGENSTRAT” and PLINK Multidimensional scaling analysis (MDS) to investigate the structure of the first stage GWAS samples. Based on the distribution of the observed –log_10_(trend test P-value) for the whole-genome SNPs against the theoretical distribution of expected –log_10_(trend test P-value), the quantile-quantile (Q-Q) plot was produced to imply the potential existence of HBV-associated genetic variants.

In the evaluation of the cumulative effects of the risk alleles identified, we calculated the ORs and 95% CIs using logistic regression models with the median number of total risk alleles used as the reference group for the total 2688 individuals.

To assess the association between the clinical characteristics and the SNPs, the three clinical groups (PNALT, CHB and HCC) were investigated in the case-only analysis. Each of the three groups was coded as a binary trait and was tested for SNP associations using logistic regression models under assumptions of three modes of inheritance and with age adjustment. In the restrained samples who received any one of three anti-HBV treatment courses, the relationship of the SNP genotypes and the therapeutic response was evaluated using the Fisher’s exact test in each treatment group and the Cochran-Mantel-Haenszel (CMH) test after controlling for treatment. In logistic regression analysis, the additive effect of risk alleles on the therapeutic response was assessed in each treatment group with age adjustment and in the full sample with age and treatment adjustment. In addition, association of inferred haplotype categories with therapeutic response was analyzed to evaluate the combined effects of multiple causal alleles and loci.

## Results

### Genome Wide Association Study and Replication

In the first-stage GWAS scan, the Illumina HumanHap610 beadchips annotated 598,821 SNPs in the cases and the Illumina HumanHap550 beadchips annotated 560,184 SNPs in the controls. The gene chips covered 547,435 SNPs in common. After the quality control filtering, a total of 456,262 SNPs on the 22 autosomal chromosomes were obtained and then used in our initial discovery of GWAS. The population structure (Figures S1 (A) and (B)) and the Q-Q plot (Figure S2) using the whole genome genotype data of 321 cases and 304 controls indicated no evidence of population stratification between the HBV cases and the controls (inflation factor λ = 1.002).

**Figure 1:**
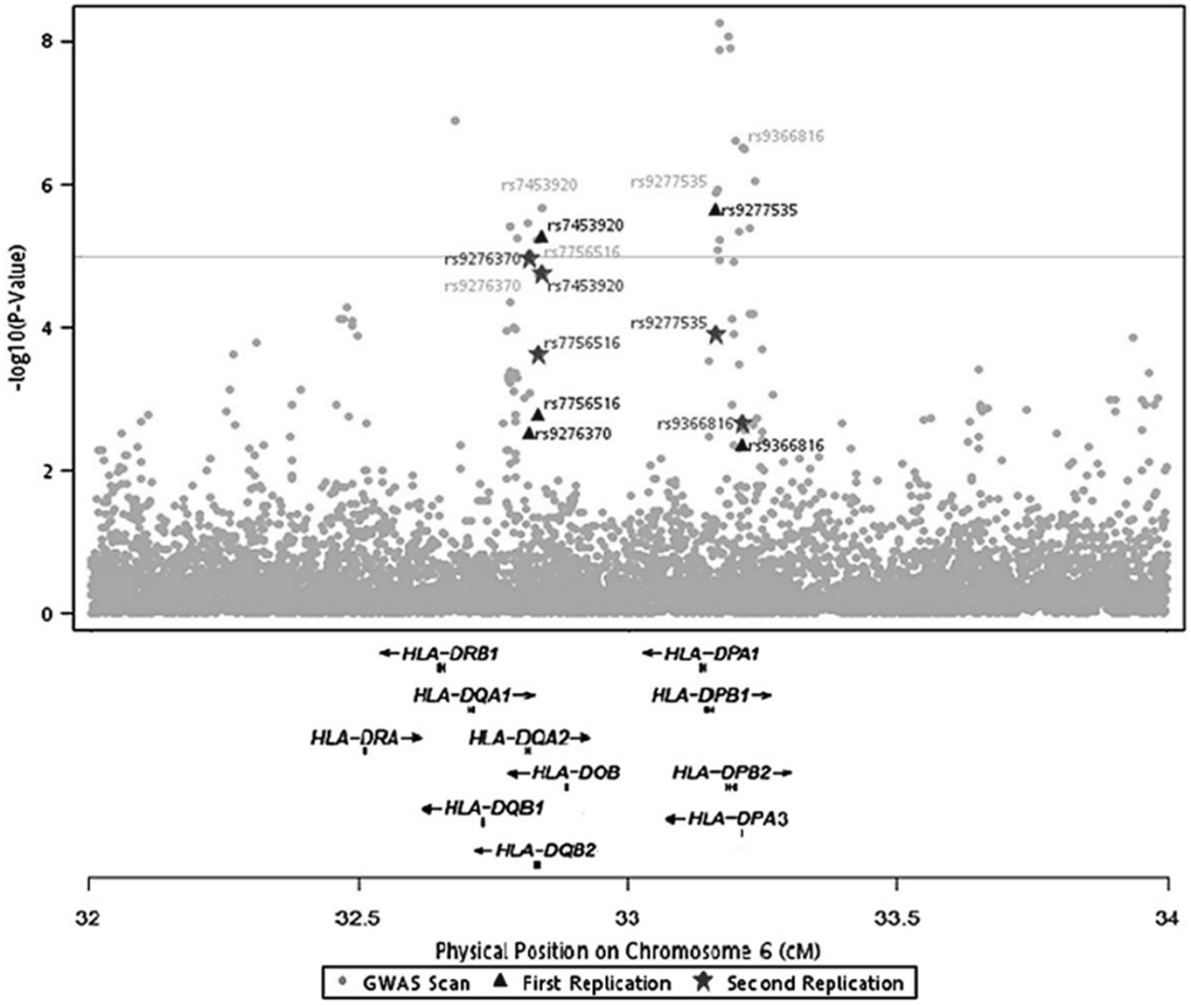
GWAS results. Multi-stage signals of association with HBV infection in the HLA region.

A total of 61 SNPs which reached the significance level of 10^−5^ were found in the whole genome (Table S1 and Figure S3). After eliminating the SNPs which failed to be validated by Taqman genotyping, we focused our study on thirty-eight SNPs on chromosome 6 (P<10^−5^ in any of the three tests; Figure 1 and Table S1). Based on the Linkage Disequilibrium (LD) block and the trend test P-value, fifteen SNPs were selected for the first replication in an independent cohort of 646 cases and 345 controls (Table S2). Seven SNPs showed significant association with P<0.01 in the first replication.

Two SNPs rs9277554 and rs2051549 were not considered further due to their close proximity to rs9277535 and rs7453920. The other five significantly associated SNPs (rs9276370, rs7756516, rs7453920, rs9277535 and rs9366816) in the first replication were selected for the second replication in an independent cohort with 656 cases and 416 controls.

In the second replication, all the five SNPs rs9276370 (P = 2.09×10^−5^, OR = 1.87), rs7756516 (P = 3.26×10^−4^, OR = 1.69), rs7453920 (P = 2.15×10^−5^, OR = 2.08), rs9277535 (P = 8.72×10^−5^, OR = 1.47) and rs9366816 (P = 7.94×10^−4^, OR = 1.35) remained significantly associated with HBV persistence after age adjustment (Table 2).

**Table 2:** Summary of results for the three-stage association study.

SNP	PhysicalPosition^a^	AnnotatedGene	Allele(A/B)^b^	Stage	Case			MAF	Control			MAF	P-value^c^	OR (95% CI)^c^
					AA	AB	BB		AA	AB	BB			
rs9276370	Chr6: 32815273	HLA-DQA2	G/**T**	GWAS	0.003	0.137	0.860	0.072	0.020	0.260	0.720	0.150	3.57×10^−5^	2.25 (1.53–3.31)
				1st replication	0.006	0.1271	0.867	0.070	0.012	0.194	0.793	0.110	2.90×10^−3^	1.65 (1.19–2.30)
				2nd replication	0.006	0.157	0.857	0.085	0.015	0.260	0.725	0.145	2.09×10^−5^	1.87 (1.40–2.50)
				Combined	0.006	0.141	0.853	0.076	0.015	0.240	0.745	0.135	1.90×10^−12^	1.95 (1.62–2.34)
rs7756516	Chr6: 32831895	HLA-DQB2	C/**T**	GWAS	0.012	0.128	0.860	0.076	0.020	0.280	0.701	0.160	1.97×10^−5^	2.24 (1.55–3.23)
				1st replication	0.008	0.124	0.867	0.071	0.012	0.204	0.784	0.114	1.35×10^−3^	1.71 (1.23–2.37)
				2nd replication	0.007	0.176	0.818	0.094	0.017	0.260	0.723	0.147	3.26×10^−4^	1.69 (1.27–2.25)
				Combined	0.009	0.146	0.846	0.081	0.016	0.248	0.736	0.140	1.48×10^−11^	1.87 (1.56–2.25)
rs7453920*	Chr6: 32837990	HLA-DQB2	A/**G**	GWAS	0.003	0.084	0.913	0.045	0.016	0.207	0.776	0.120	9.24×10^−6^	2.77 (1.77–4.35)
				1st replication	0.003	0.093	0.904	0.050	0.015	0.181	0.804	0.105	1.02×10^−5^	2.22 (1.56–3.16)
				2nd replication	0.002	0.109	0.890	0.056	0.010	0.192	0.798	0.106	2.15×10^−5^	2.08 (1.48–2.91)
				Combined	0.003	0.098	0.900	0.051	0.013	0.193	0.794	0.110	6.66×10^−15^	2.31 (1.87–2.85)
rs9277535*	Chr6: 33162839	HLA-DPB1	A/**G**	GWAS	0.062	0.343	0.595	0.234	0.106	0.503	0.391	0.358	1.80×10^−6^	1.87 (1.45–2.42)
				1st replication	0.071	0.357	0.572	0.249	0.142	0.428	0.431	0.355	3.64×10^−6^	1.61 (1.32–1.98)
				2nd replication	0.076	0.372	0.553	0.262	0.125	0.431	0.443	0.341	8.72×10^−5^	1.47 (1.21–1.78)
				Combined	0.071	0.360	0.569	0.251	0.125	0.451	0.424	0.350	4.87×10^−14^	1.59 (1.41–1.79)
rs9366816*	Chr6: 33212153	HLA-DPA3	**C**/T	GWAS	0.393	0.442	0.165	0.386	0.188	0.566	0.247	0.470	1.07×10^−6^	1.80 (1.42–2.27)
				1st replication	0.353	0.456	0.191	0.419	0.247	0.531	0.222	0.488	5.45×10^−3^	1.31 (1.08–1.58)
				2nd replication	0.353	0.448	0.199	0.423	0.260	0.494	0.246	0.493	7.94×10^−4^	1.35 (1.13–1.61)
				Combined	0.361	0.450	0.189	0.414	0.235	0.526	0.239	0.498	2.58×10^−10^	1.43 (1.28–1.60)

*Used in risk score calculation.

a Genome Build 36.3.

b Risk alleles were in bold.

c P-values, ORs and CIs were calculated based on the additive effect model with age adjustment.

The combined analysis of total series revealed consistent results as in the single stages. The most significant association was conferred by rs7453920 (P = 6.66×10^−15^, OR = 2.31) on HLA-DQB2 and rs9277535 on HLA-DPB1 (P = 4.87×10^−14^, OR = 1.59).

### Pairwise LD Patterns in the Five Associated SNPs and Haplotype Association

**Figure 2:**
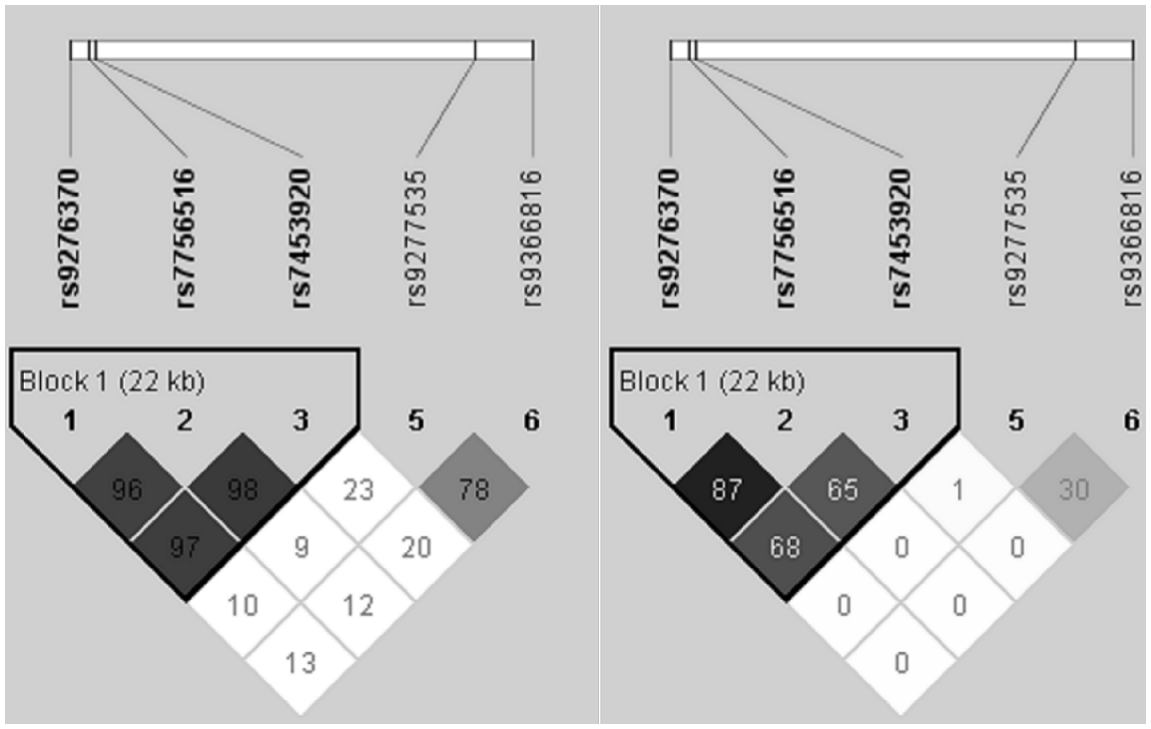
LD patterns of the 5 SNPs. LD patterns based on the D’ (left side) and r^2^ (right side) measures of the five associated SNPs for 625 individuals in the GWAS scan. The LD block built up by the rs9276370, rs7756516 and rs7453920 suggest that they were significantly associated with each other. The rs9366816 and rs9277535 SNPs were independent from each other and from the other three SNPs.

The pair wise LD of the five associated SNPs was calculated for the 625 individuals in the GWAS scan. The haplotype block was generated from the Haploview software (Figure 2). The LD block built up by the three SNPs rs9276370, rs7756516 and rs7453920 suggested that they might not contribute independently to the disease risk. The rs9277535 and rs9366816 were independent from the other three SNPs mentioned above, and they showed weak association with each other (r^2^ = 0.30). Hence we re-evaluated the association of each one of the five SNPs using logistic regression analysis adjusted for the other specified SNP and age in the combined samples. As a result, the rs7453920 remained significant after controlling for the effect of nearby rs9276370 and rs7756516 (Table S3 P-value = 5.49×10^−4^ and 4.06×10^−4^, respectively). The additional rs9277535 on HLA-DPB1 and rs9366816 near HLA-DPA3 were also associated with persistent HBV infections independently (P-value = 9.09×10^−7^). In the haplotype analysis, three major haplotypes “T-T-G-G-T” (48.96%), “T-T-G-A-T” (21.45%) and “T-T-G-G-C” (16.11%) formed by the five SNPs were specified (Table 3). After examining haplotype-disease association using logistic regression models, the “T-T-G-G-T” haplotype appeared to confer evident susceptibility to persistent HBV infection (52.83% in cases vs. 43.05% in controls; P-value = 1.48×10^−12^, OR = 1.49, 95% CI: 1.33–1.66 after age adjustment). We also found that the “T-T-G-A-T” haplotype had protective effect from developing the disease (19.69% in cases vs. 24.13% in controls; P-value = 1.17×10^−4^, OR = 0.77, 95% CI: 0.68–0.88 after age adjustment). When estimating analyzing the haplotypes made up of four SNPs excluding rs9366816, the opposite effects of the rs9277535 G and A alleles contributing to persistent HBV infection appeared to be more prominent (Table S5).

**Table 3:** Haplotype association of the 5 risk SNPs rs9276370, rs7756516, rs7453920, rs9277535 and rs9366816 with persistent HBV infection.

Haplotype	Estimated Frequency (%)			Logistic regression		Logistic regression adjusted for age	
	Case	Control	All	P-value	OR (95% CI)	P-value	OR (95% CI)
	(N = 1623)	(N = 1065)	(N = 2688)				
T-T-G-G-T	52.83%	43.05%	48.96%	2.47×10^−12^	1.48 (1.33–1.65)	1.48×10^−12^	1.49 (1.33–1.66)
T-T-G-A-T	19.69%	24.13%	21.45%	1.05×10^−4^	0.77 (0.68–0.88)	1.17×10^−4^	0.77 (0.68–0.88)
T-T-G-G-C	16.88%	14.93%	16.11%	0.0569	1.16 (1.00–1.35)	0.0689	1.15 (0.99–1.34)
All other	10.60%	17.89%	13.49%	3.51×10^−14^	0.54 (0.47–0.64)	2.39×10^−14^	0.54 (0.46–0.63)

### Clinical Significance of the SNPs

Among the 1623 HBsAg carriers, the SNP rs9276370 showed a lower TT genotype frequency in the PNALT group than in the other two groups (0.825 vs. 0.865; trend test p value = 0.0241; Table 4). The test for additive effects of the T alleles reached significance with P = 0.0376 (OR = 0.73; 95% CI: 0.55–0.96).

**Table 4:** Association analysis of rs9276370 with clinical classifications in HBsAg positive carriers.

Clinical characteristic	Genotype Frequency for the group of interest			MAF	Genotype Frequency for the other two groups			MAF	Trend test P-value	Testing for mode of inheritance	P-value^a^	OR (95% CI)^a^
	GG	GT	TT		GG	GT	TT					
PNALT	0.011	0.164	0.825	0.093	0.004	0.132	0.865	0.069	0.0241	Additive	0.0376	0.73 (0.55–0.96)
										Dominant	0.0667	1.32 (0.98–1.78)
										Recessive	0.0947	3.09 (0.82–11.63)
CHB	0.000	0.146	0.854	0.073	0.011	0.136	0.853	0.079	0.5247	Additive	0.3746	1.13 (0.87–1.47)
										Dominant	0.7610	0.96 (0.72–1.27)
										Recessive	0.9719	N/A
HCC	0.011	0.101	0.888	0.062	0.004	0.153	0.843	0.080	0.0923	Additive	0.2134	1.25 (0.88–1.76)
										Dominant	0.0903	1.38 (0.95–2.00)
										Recessive	0.0896	3.25 (0.83–12.70)

a ORs and CIs were calculated by setting the T allele as the reference with age adjustment.

Those in the PNALT group were less likely to carry rs7756516-T alleles compared to the other two more aggressive liver disease groups (CHB and HCC, Table S4). The frequency of the TT genotype was lower in the PNALT group than in the other two groups (0.815 vs 0.865; additive genetic effects P-value = 0.0249; OR = 0.74, 95% CI: 0.56–0.96; Table S4).

### Therapeutic Response

Among the CHB group, we reviewed the charts retrospectively and focused on the patients who received a course of the anti-HBV therapy. A variety of regimens developed in the past 20 to 30 years. We only considered the patients who received three widely used regimens: (1) lamivudine 100 mg daily, which has been used widely since its availability in early 2000 [19]; (2) entevavir 0.5 mg daily, soon replacing lamivudine as the most widely used oral antiviral agent accredited to its high potency of antiviral effect and low rate of drug resistance [20]; and (3) pegylated interferon α-2a 135–180 mcg weakly for one year [21]. Most of the patients received free therapies according to the nationwide health insurance policy. The regimen initially covered a one-year therapy only but changed to an up to 4–year regimen in recent years instead [22]–[24]. Therefore, we excluded those who received lamivudine or entecavir for <1 year or >4 year, and those who received pegylated interferon α-2a for <1 year. A total of 226 patients were included for this analysis. Those patients with HBV DNA<10^4^ cps/mL with normal ALT at the 1-year post-treatment and then remained persistent normal ALT, or transient ALT elevation to <2 times the upper limit of normal during follow-up were defined as sustained responders [25]–[26]. Those patients with ALT>2 times the upper limit of normal or HBV DNA>10^4^ during the post-treatment follow-up period were defined as non-sustained responders.

The treatment naive rate was highest in the lamivudine group (69.8%), followed by 40.6% in the entecavir group and 39.5% in the pegylated interferon α-2a group (P<10^−4^; data not shown). The mean age at treatment was older in the entecavir group than in the other two groups (P = 10^−4^, Table 5). In the 226 cases, the frequency of GG and GT genotypes in the non-sustained responders was only 12.43% compared to 26.32% in the sustained responders (Fisher’s exact P = 0.0121 and CMH associated P = 0.0190). Similar trends were found in the treatment groups of lamivudine and pegylated interferon α-2a, but not in the entecavir group. Furthermore, in logistic regression analysis, the association of rs9276370 G alleles revealed significant additive effect on therapeutic response after adjusted for age and treatment (P = 0.0138, OR = 2.51, 95% CI: 1.21–5.22, Table 5). In estimating the haplotypes composed of two risk SNPs rs9276370 and rs7756516, two major haplotypes “TT” (90.93%) and “GC” (7.74%) were obtained (Table S6). The “G-C” haplotype had positive effect on sustained therapeutic response (P-value = 0.0132, OR = 2.49, 95% CI: 1.21–5.11 after adjusting for age at treatment); while the majority “T-T” haplotype showed negative effect on sustained therapeutic response (P-value = 0.0262, OR = 0.46, 95% CI: 0.23–0.91 after adjusting for age at treatment) as given in Table S6. The result indicated the potential use of the “T-T” and “G-C” for distinguishing the two types of therapeutic responders, although further replication studies with larger sample size should definitely be carried out to validate their effect.

**Table 5:** Association of the rs9276370 genotypes with therapeutic response in three treatment groups.

Treatmentgroup	Age at treatment(year) Mean ± SD	Post-treatmentfollow-up (month) Mean ± SD	rs9276370Genotype	Therapeuticresponse		Fisher’sexact p-value	Logisticregression^b^	
				Non-sustainedN (%)	Sustained N (%)		P-value^b^	OR (95% CI)^b^
Lamivudine 100 mg/dailyfor 1–4 years	45.49±10.05	77.78±26.78	GG	0 (0)	1 (3.57)	0.0074	0.0037	5.41 (1.73–16.95)
			GT	7 (7.69)	7 (25.00)			
			TT	84 (92.31)	20 (71.43)			
Entecavir 0.5 mg/dailyfor 1–4 years	51.58±8.65	38.34±15.90	GT	11 (23.40)	3 (17.65)	0.7425	0.5954	0.68 (0.16–2.86)
			TT	36 (76.60)	14 (82.35)			
Pegasys 135–180 mcg/weaklyfor 1 year	45.95±8.11	39.02±20.30	GT	3 (9.68)	4 (33.33)	0.0814	0.0676	5.44 (0.88–33.48)
			TT	28 (90.32)	8 (66.67)			
Total	47.30±9.67	59.24±30.13	GG	0 (0)	1 (1.75)	0.0121(CMH P^a^ = 0.0190)	0.0138	2.51 (1.21–5.22)
			GT	21 (12.43)	14 (24.56)			
			TT	148 (87.57)	42 (73.68)			

a CMHP: The Cochran-Mantel-Haenszel (CMH) p-value for general association was tested for the relationship between the rs9276370 genotypes and the therapeutic response after controlling for treatment.

b The additive G allele effect on the therapeutic response (sustained vs. non-sustained) was evaluated in logistic regression analysis. For each of the three treatment groups, the P-values, ORs and CIs were adjusted for age, whereas for the entire sample, the statistics were adjusted for age and treatment.

## Discussion

From a total of 1065 male controls and 1623 male HBsAg carriers, this multi-stage GWAS study identified that three SNPs within HLA-DPB1 (rs9277535), HLA-DQB2 (7453920) and HLA-DPA3 (rs9366816) loci were independently associated with persistence HBV infection in a male Taiwanese Han Chinese population. The former two SNP associations were also reported in previous Japanese and Mainland Chinese GWAS studies [11], [12], [27]. Our findings confirmed that HLA-DP and -DQ loci played important roles in the development of persistent HBV infection.

A study from Mainland China revealed that HLA-DPA1 was highly associated with persistent HBV infection in Northern Han Chinese; while HLA-DPB1 was associated with the Southern minorities [28]. Taiwan Han Chinese mainly migrated from southern China [29]. Thus we did not identify SNPs in HLA-DPA1 in the GWAS scan. In addition to HLA-DP and HLA-DQ, a recent publication from Hu et al. (2013) reported that two novel SNPs rs3130542 (near HLA-C) and rs4821116 (in UBE2L3) were associated with persistent HBV infection in central China [27]. However, both of the SNPs did not show significant signals in our screening stage.

The rs9277535 in 3′UTR of HLA-DPB1 showed significant association with persistent HBV replication in our as well as other series (Table 2). The results of the haplotype analysis implied the crucial role that the rs9277535 SNP might play in the HBV persistence and viral clearance. In both of our haplotype analysis composed of five and four (including and excluding rs9366816), the individuals with the “T-T-G-G-T” or “T-T-G-G” haplotype were more likely to be persistent HBsAg carriers compared to those without the haplotypes (OR = 1.49 and 1.67 respectively); whereas those with the “T-T-G-A-T” or “T-T-G-A” haplotype were more likely to clear the HBV virus (Table 4 and S5).

In a study of European and Africa-American populations, rs9277534 rather than rs9277535 in the HLA-DPB1 3′UTR region was associated with HBV recovery [30]. In their flow cytometry and mRNA analysis, the HLA-DP expression level was higher with the GG genotype than with the other genotypes. The higher HLA-DP expression might promote mechanisms of high HBV clearance in the controls [30], [31].

The SNP rs7453920 (HLA-DQB2) showed significant association with persistent HBV infection but did not associate with clinical outcome [32]. The SNP rs7756516 on 3′UTR of HLA-DQB2 identified in our cohort also showed significant association between the PNALT group and the other two more aggressive disease groups (CHB and HCC) (Table S4, additive effect P = 0.0249; OR = 0.74). There is a lower prevalence of rs7756516 genotype TT in the PNALT group than in the other two groups (0.815 vs. 0.865, trend test P = 0.0134). From the NIH web site, the highest prevalence of TT genotype of rs7756516 (0.788) was reported for the Han Chinese in Metropolitan Denver (HAPMAP-CHD). Their prevalence is similar to the prevalence in our controls (0.736) and lower than that in our cases (0.846, Table 2). These findings are compatible with a high prevalence of HBsAg in Taiwan [6].

The SNP rs9276370 near the 5′ region of HLA-DQA2 was also associated with persistent HBV infection (Table 1) and clinical progression (Table 3). Together with data of HLA-DQB2, the HLA-DQA2 and HLA-DQB2 complex may participate in HBV persistent infection and disease progression. In the 226 cases who received a course of anti-HBV therapy, a higher non-sustained responder rate was found in the TT genotype of rs9276370. This is especially true in the lamivudine group (P = 0.0074) and the pegylated interferon α-2a group (P = 0.0814, Table 4). The entecavir group did not show this trend. The mean age at treatment in the lamivudine group was younger and had been followed for a longer period (P = 10^−4^, Table 5). There was also a higher prevalence of treatment naive cases (69.8%) than the other two groups (40.6% and 39.5%; P<10^−4^). Whether these differences may explain the therapeutic difference between Lamivudine and entecavir groups remain uncertain. Entecavir is a strong nucleoside analog that may suppress HBV replication completely [33]. The low HBV protein expression may decrease the impact of antigen presentation by HLA. The results of our haplotype analysis indicated the important role that HLA-DQA2 rs9276370 and HLA-DQB2 rs7756516 might play in therapeutic response. The “T-T” haplotype composed of rs9276370 and rs7756516 was associated with non-sustained therapeutic response (P = 0.0262, Table S6); while the “G-C” haplotype was associated with sustained therapeutic response (P = 0.0132; OR = 2.49). The “T-T” and “G-C” haplotypes have the potential for predicting the two types of therapeutic responders, although the sample size of the current analysis was too small and further replication studies should be carried out to validate the combined effects of these two SNPs.

Recently, Pan et al. identified that four tagging SNPs rs477515, rs28366298, rs3763316 and rs13204672 showed significant associations with non-response to hepatitis B vaccination [34]. The SNP rs477515 (located ∼12 kb upstream of HLA-DRB1) as well as rs2516049 achieved the significance level in our initial screening stage, but we failed to validate them due to multiple repetitive sequences near these SNPs.

The rs9366816 near HLA-DPA3 was associated with persistent HBV infection (Figure 2 and Table S3). The rs9366816 is close to HLA-DPB2 as well. The information about these two HLA class II pseudogenes is limited [35]. The SNP rs9366816 in HLA-DPA3 is not in LD to with all the other associated SNPs except for rs9277535 (r^2^ = 0.30; Figure 2), which implies its independent contribution to the disease susceptibility.

We concluded that HLA-DPB1, HLA-DQA2 and HLA-DQB2 are associated with persistent HBV infection in male Han Taiwanese. HLA-DQA2 and -DQB2 complex is associated with the clinical progression and therapeutic response of chronic HBV infection.

## Supporting Information

Figure S1 **Population structure.**
(DOCX)

Figure S2 **Quantile-Quantile (Q-Q) plot.**
(DOCX)

Figure S3 **Manhattan plot for the GWAS of HBV infection.**
(DOCX)

Table S1 **List of 61 SNPs which reached the significance level of 10^−5^ at the first stage GWAS scan.**
(DOCX)

Table S2 **Summary of SNPs examined in the first replication.**
(DOCX)

Table S3 **Logistic regression analysis for the association of the each SNP in persistent HBV infection after controlling other SNPs and age in the combined samples.**
(DOCX)

Table S4 **Association analysis of rs7756516 with clinical classifications in HBsAg positive carriers.**
(DOCX)

Table S5 **Haplotype association of the 5 risk SNPs rs9276370, rs7756516, rs7453920 and rs9277535 with persistent HBV infection.**
(DOCX)

Table S6 **Haplotype association of 2 SNPs rs9276370 and rs7756516 with therapeutic response.**
(DOCX)
