# Supplementary figures and images for: A Genome-Wide Association Study on Chronic HBV Infection and Its Clinical Progression in Male Han-Taiwanese
Source: PLoS One. 2014 Jun 18;9(6):e99724. doi: 10.1371/journal.pone.0099724 (PMC4062466; doi:10.1371/journal.pone.0099724)

**Figure S1 Population structure**


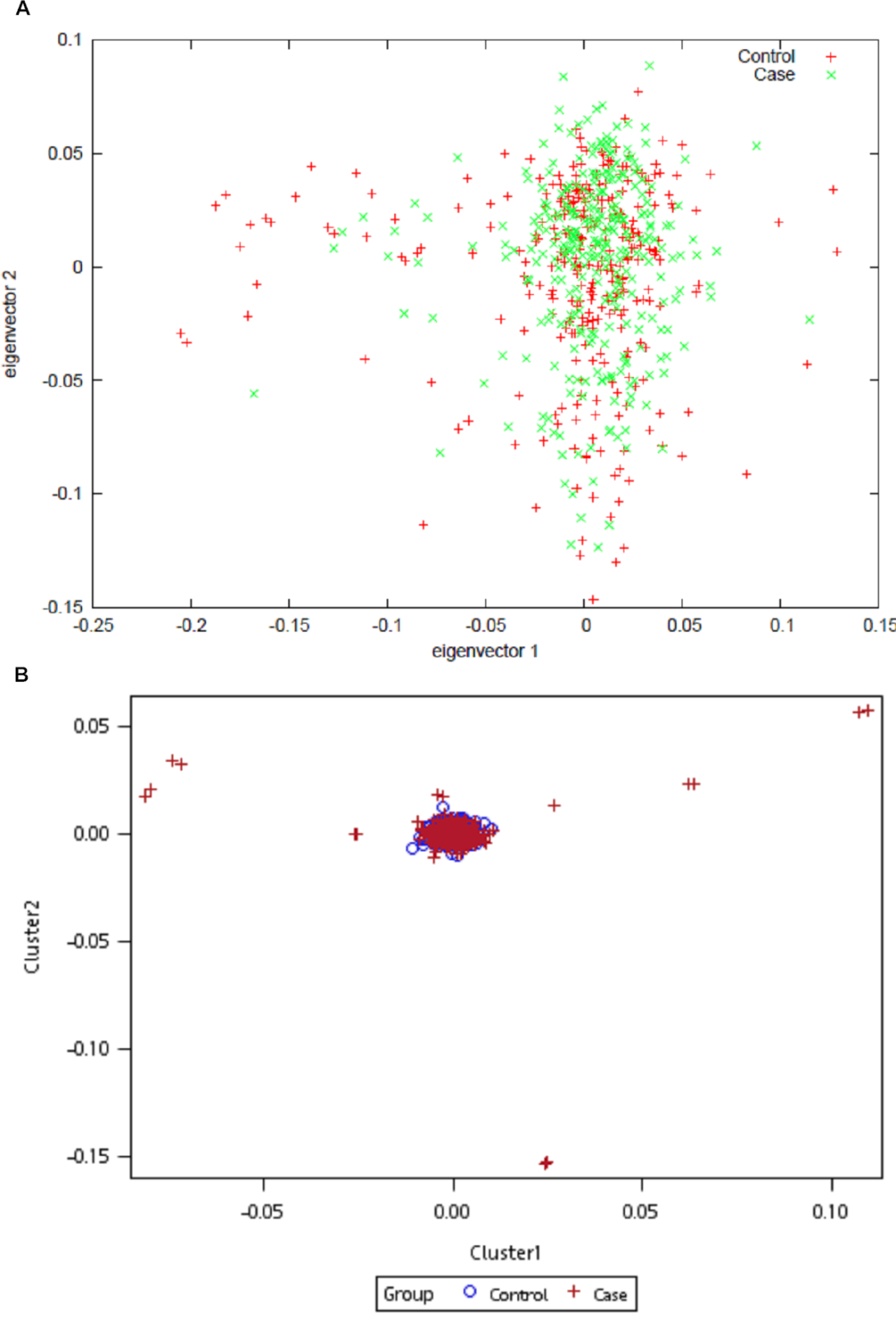

Supplement: Figure S1 — Population structure. (DOCX) [file pone.0099724.s001.docx]

**Figure S2 Quantile-Quantile (Q-Q) plot**


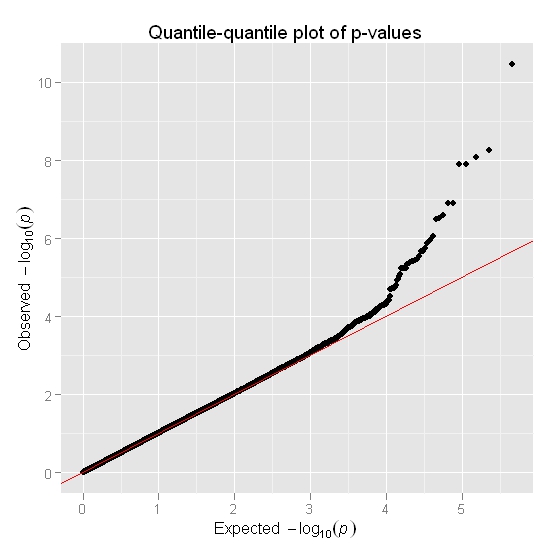

Supplement: Figure S2 — Quantile-Quantile (Q-Q) plot. (DOCX) [file pone.0099724.s002.docx]

**Figure S3 Manhattan plot for the GWAS of HBV infection**


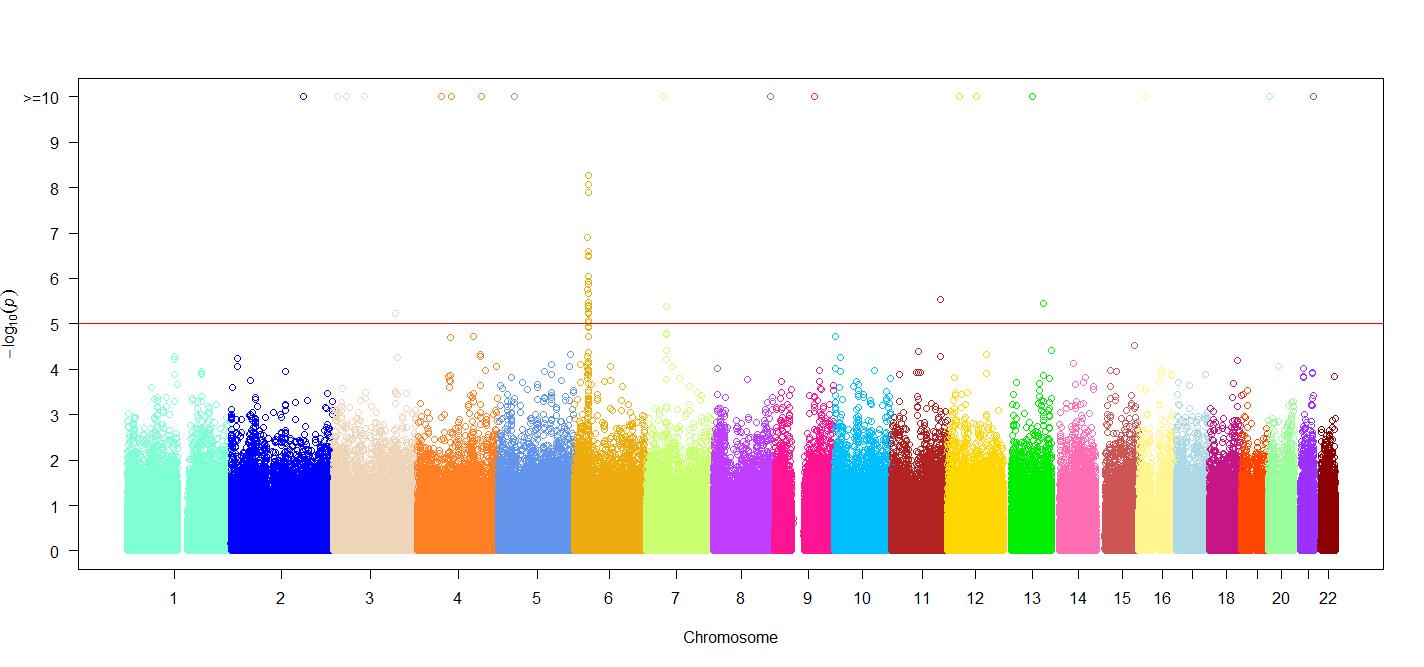

Supplement: Figure S3 — Manhattan plot for the GWAS of HBV infection. (DOCX) [file pone.0099724.s003.docx]
