# Supplementary material for: A Genome-Wide Association Study on Chronic HBV Infection and Its Clinical Progression in Male Han-Taiwanese
Source: PLoS One. 2014 Jun 18;9(6):e99724. doi: 10.1371/journal.pone.0099724 (PMC4062466; doi:10.1371/journal.pone.0099724)
Supplement: Table S1 — List of 61 SNPs which reached the significance level of 10−5 at the first stage GWAS scan. (DOCX) [file pone.0099724.s004.docx]

**Table S1 List of 61 SNPs which reached the significance level of 10^-5^ at the first stage GWAS scan**

| **SNP** | **Allele** | **Chromosome** | **Physical position^a^** | **Nearest gene** | **GWAS** | | |
| --- | --- | --- | --- | --- | --- | --- | --- |
|  |  |  |  |  | **–log_10_(trend test *P*-value)** | **–log_10_(allele test *P*-value)** | **–log_10_(genotype test *P*-value)** |
| rs1549343 | CT | 2 | 170802031 | MYO3B | 48.560 | 57.532 | 54.412 |
| rs446975 | GT | 3 | 9192383 | SRGAP3 | 35.551 | 40.407 | 37.373 |
| rs6797523 | CT | 3 | 30300054 | . | 78.268 | 78.268 | 78.268 |
| rs1447826 | AG | 3 | 74801448 | . | 78.268 | 78.268 | 78.268 |
| rs986173 | AG | 3 | 147884376 | . | 5.238 | 4.973 | 5.310 |
| rs2726724 | GT | 4 | 56227094 | . | 35.145 | 39.193 | 34.250 |
| rs1455311 | CT | 4 | 80183611 | . | 78.268 | 78.268 | 78.268 |
| rs361147 | AC | 4 | 153110013 | AC097375.3 | 31.065 | 34.324 | 30.687 |
| rs10053502 | CT | 5 | 40014929 | . | 42.201 | 49.164 | 44.002 |
| rs2076530 | AG | 6 | 32471794 | BTNL2 | 5.747 | 6.085 | 5.664 |
| rs477515 | CT | 6 | 32677669 | LOC100507709, HLA-DRB1 | 6.897 | 6.552 | 6.306 |
| rs2516049 | AG | 6 | 32678378 | LOC100507709, HLA-DRB1 | 6.897 | 6.552 | 6.306 |
| rs9275371 | CT | 6 | 32776274 | HLA-DQA2 | 3.313 | 3.243 | 5.276 |
| rs9275374 | CT | 6 | 32776504 | HLA-DQA2 | 3.313 | 3.243 | 5.276 |
| rs9275388 | CT | 6 | 32777062 | MTCO3P1 | 3.268 | 3.203 | 5.184 |
| rs9275390 | CT | 6 | 32777134 | MTCO3P1 | 3.313 | 3.243 | 5.276 |
| rs9275393 | AG | 6 | 32777417 | MTCO3P1 | 3.313 | 3.243 | 5.276 |
| rs2647050 | CT | 6 | 32777745 | MTCO3P1 | 5.409 | 5.449 | 5.196 |
| rs9275406 | GT | 6 | 32777933 | MTCO3P1 | 3.313 | 3.243 | 5.276 |
| rs9275407 | GT | 6 | 32778015 | MTCO3P1 | 3.313 | 3.243 | 5.276 |
| rs9275418 | AG | 6 | 32778222 | MTCO3P1 | 3.313 | 3.243 | 5.276 |
| rs2856718 | AG | 6 | 32778233 | MTCO3P1 | 5.409 | 5.449 | 5.196 |
| rs9275427 | CT | 6 | 32778893 | MTCO3P1 | 3.216 | 3.151 | 5.117 |
| rs9275428 | AG | 6 | 32778956 | MTCO3P1 | 3.398 | 3.344 | 5.160 |
| rs9275439 | CT | 6 | 32779499 | MTCO3P1 | 3.313 | 3.243 | 5.276 |
| rs3873444 | AG | 6 | 32790702 | . | 5.245 | 5.098 | 5.245 |
| rs2859078 | CT | 6 | 32810427 | HLA-DQB3 | 5.469 | 5.349 | 5.469 |
| rs9276370 | GT | 6 | 32815273 | HLA-DQA2 | 5.030 | 4.992 | 4.266 |
| rs10807113 | AC | 6 | 32830164 | HLA-DQB2 | 5.223 | 5.329 | 5.089 |
| rs7756516 | CT | 6 | 32831895 | HLA-DQB2 | 5.223 | 5.329 | 5.089 |
| rs7453920 | AG | 6 | 32837990 | HLA-DQB2 | 5.675 | 5.875 | 4.943 |
| rs2051549 | CT | 6 | 32838064 | HLA-DQB2 | 5.675 | 5.875 | 4.943 |
| rs9277535 | AG | 6 | 33162839 | AL645931.7, HLA-DPB1 | 5.875 | 5.803 | 5.688 |
| rs9277554 | CT | 6 | 33163516 | AL645931.7, HLA-DPB1 | 5.941 | 5.893 | 5.689 |
| rs9277565 | CT | 6 | 33164875 | AL645931.7, HLA-DPB1 | 5.078 | 5.131 | 4.588 |
| rs10484569 | AG | 6 | 33166930 | AL645931.7, HLA-DPB1 | 8.259 | 8.393 | 9.091 |
| rs3128917 | GT | 6 | 33167974 | AL645931.7, HLA-DPB1 | 4.951 | 5.014 | 4.433 |
| rs2281388 | CT | 6 | 33168096 | AL645931.7, HLA-DPB1 | 7.891 | 7.876 | 8.807 |
| rs3117222 | AG | 6 | 33168927 | AL645931.7, HLA-DPB1 | 5.229 | 5.370 | 4.650 |
| rs6457713 | CT | 6 | 33185754 | AL645940.4, COL11A2P | 8.080 | 8.233 | 8.966 |
| rs9380343 | CT | 6 | 33187144 | AL645940.4, COL11A2P | 7.899 | 7.906 | 8.846 |
| rs4713607 | AG | 6 | 33198814 | AL645940.4 | 6.600 | 6.512 | 6.809 |
| rs3117008 | CT | 6 | 33204252 | HLA-DPA3 | 5.331 | 5.310 | 4.902 |
| rs9366816 | CT | 6 | 33212153 | HLA-DPA3 | 6.508 | 6.434 | 6.998 |
| rs13437000 | CT | 6 | 33213042 | HLA-DPA3 | 6.491 | 6.351 | 6.814 |
| rs721394 | AG | 6 | 33225796 | . | 5.393 | 5.235 | 5.958 |
| rs9277920 | AG | 6 | 33233703 | COL11A2 | 6.045 | 5.857 | 5.895 |
| rs3735135 | CT | 7 | 39994009 | CDC2L5 | 52.877 | 60.461 | 52.050 |
| rs2470996 | AC | 7 | 47399194 | TNS3 | 4.798 | 5.194 | 4.189 |
| rs11764365 | AG | 7 | 47414066 | TNS3 | 5.374 | 5.617 | 4.849 |
| rs1036819 | AC | 8 | 135681127 | ZFAT | 78.268 | 78.268 | 78.268 |
| rs4909376 | AC | 8 | 138673540 | . | 0.848 | 0.847 | 5.467 |
| rs430794 | AC | 9 | 92892636 | . | 37.071 | 42.553 | 37.964 |
| rs3802871 | GT | 11 | 117281858 | TMPRSS13 | 5.532 | 5.355 | 5.032 |
| rs1975920 | GT | 12 | 27627253 | PPFIBP1 | 78.268 | 78.268 | 78.268 |
| rs7137203 | CT | 12 | 67834426 | . | 10.466 | 10.152 | 10.466 |
| rs9572312 | CT | 13 | 69406164 | KLHL1 | 78.268 | 78.268 | 78.268 |
| rs7991937 | AG | 13 | 93607727 | GPC6 | 5.443 | 5.242 | 4.663 |
| rs11075260 | GT | 16 | 15192528 | . | 41.214 | 48.091 | 42.017 |
| rs1810636 | GT | 20 | 2602925 | FASTKD5 | 76.243 | 78.268 | 78.268 |
| rs2236479 | AG | 21 | 45743560 | SLC19A1, COL18A1 | 78.268 | 78.268 | 78.268 |
