# Supplementary material for: A Genome-Wide Association Study on Chronic HBV Infection and Its Clinical Progression in Male Han-Taiwanese
Source: PLoS One. 2014 Jun 18;9(6):e99724. doi: 10.1371/journal.pone.0099724 (PMC4062466; doi:10.1371/journal.pone.0099724)
Supplement: Table S2 — Summary of SNPs examined in the first replication. (DOCX) [file pone.0099724.s005.docx]

**Table S2 Summary of SNPs examined in the first replication**

| **SNP** | **Allele** | **Chromosome** | **Physical position^a^** | **Nearest gene** |  | **1^st^ replication** | |
| --- | --- | --- | --- | --- | --- | --- | --- |
|  |  |  |  |  |  | **Trend test *P*-value** | **Genotyping method** |
| rs1455311 | CT | 4 | 80183611 | LOC100505875 |  | 0.3337 | Taqman |
| rs2076530 | AG | 6 | 32471794 | BTNL2 |  | 0.2603 | Taqman |
| rs477515 | CT | 6 | 32677669 | LOC100507709, HLA-DRB1 |  | Failed | Taqman |
| rs2647050 | CT | 6 | 32777745 | MTCO3P1 |  | 0.0987 | Taqman |
| rs2856718 | AG | 6 | 32778233 | MTCO3P1 |  | 0.1039 | Taqman |
| rs9276370 | GT | 6 | 32815273 | HLA-DQA2 |  | 0.0030 | Taqman |
| rs10807113 | AC | 6 | 32830164 | HLA-DQB2 |  | 0.2802 | Sequenom |
| rs7756516 | CT | 6 | 32831895 | HLA-DQB2 |  | 0.0017 | Sequenom |
| rs7453920 | AG | 6 | 32837990 | HLA-DQB2 |  | 5.28 x 10^-6^ | Taqman |
| rs2051549 | CT | 6 | 32838064 | HLA-DQB2 |  | 0.0011 | Sequenom |
| rs9277535 | AG | 6 | 33162839 | AL645931.7, HLA-DPB1 |  | 2.26 x 10^-6^ | Taqman |
| rs9277554 | CT | 6 | 33163516 | AL645931.7, HLA-DPB1 |  | 2.20 x 10^-4^ | Taqman |
| rs10484569 | AG | 6 | 33166930 | AL645931.7, HLA-DPB1 |  | 0.1633 | Taqman |
| rs2281388 | CT | 6 | 33168096 | AL645931.7, HLA-DPB1 |  | 0.1577 | Taqman |
| rs9380343 | CT | 6 | 33187144 | AL645940.4, COL11A2P |  | 0.0983 | Taqman |
| rs9366816 | CT | 6 | 33212153 | HLA-DPA3 |  | 0.0045 | Taqman |
| rs11764365 | AG | 7 | 47414066 | TNS3 |  | 0.2688 | Taqman |
| rs3802871 | GT | 11 | 117281858 | TMPRSS13 |  | 0.2662 | Taqman |
| rs1975920 | GT | 12 | 27627253 | PPFIBP1 |  | 0.0217 | Taqman |
| rs9572312 | CT | 13 | 69406164 | KLHL1 |  | 0.5152 | Taqman |
| rs7991937 | AG | 13 | 93607727 | GPC6 |  | Failed | Taqman |
| rs1810636 | GT | 20 | 2602925 | FASTKD5 |  | 0.0655 | Taqman |
| rs2236479 | AG | 21 | 45743560 | SLC19A1, COL18A1 |  | 0.2970 | Taqman |

^a^Genome Build 36.3.
