# Supplementary material for: A Genome-Wide Association Study on Chronic HBV Infection and Its Clinical Progression in Male Han-Taiwanese
Source: PLoS One. 2014 Jun 18;9(6):e99724. doi: 10.1371/journal.pone.0099724 (PMC4062466; doi:10.1371/journal.pone.0099724)
Supplement: Table S3 — Logistic regression analysis for the association of the each SNP in persistent HBV infection after controlling other SNPs and age in the combined samples. (DOCX) [file pone.0099724.s006.docx]

**Table S3 Logistic regression analysis for the association of the each SNP in persistent HBV infection after controlling other SNPs and age in the combined samples**

|  | **SNP adjusted for*** | | | | | | | | | |
| --- | --- | --- | --- | --- | --- | --- | --- | --- | --- | --- |
|  | **rs9276370** | | **rs7756516** | | **rs7453920** | | **rs9277535** | | **rs9366816** | |
| **SNP** | **P-value** | **OR (95% CI)** | **P-value** | **OR (95% CI)** | **P-value** | **OR (95% CI)** | **P-value** | **OR (95% CI)** | **P-value** | **OR (95% CI)** |
| rs9276370 | - | - | 0.0674 | 1.56 (0.97-2.52) | 0.3867 | 1.16 (0.83-1.64) | 1.37 x 10^-11^ | 1.91 (1.58-2.30) | 1.08 x 10^-11^ | 1.91 (1.58-2.30) |
| rs7756516 | 0.3412 | 1.25 (0.79-2.00) | - | - | 0.3061 | 1.18 (0.86-1.62) | 1.99 x 10^-10^ | 1.82 (1.52-2.19) | 1.28 x 10^-10^ | 1.83 (1.52-2.20) |
| rs7453920 | 5.49 x 10^-4^ | 1.99 (1.35-2.94) | 4.06 x 10^-4^ | 1.94 (1.34-2.79) | - | - | 5.66 x 10^-13^ | 2.21 (1.78-2.74) | 1.35 x 10^-13^ | 2.24 (1.81-2.77) |
| rs9277535 | 1.07 x 10^-12^ | 1.56 (1.38-1.76) | 2.77 x 10^-13^ | 1.59 (1.40-1.80) | 1.31 x 10^-12^ | 1.56 (1.38-1.76) | - | - | 9.09 x 10^-7^ | 1.43 (1.24-1.65) |
| rs9366816 | 2.49 x 10^-13^ | 1.41 (1.26-1.58) | 2.65 x 10^-10^ | 1.44 (1.29-1.62) | 1.79 x 10^-9^ | 1.41 (1.26-1.58) | 6.83 x 10^-3^ | 1.20 (1.05-1.37) | - | - |

*For each of the five associated SNPs, the p-values, ORs and CIs were calculated using logistic regression analysis with adjustment of age and the specified SNP.
