# Supplementary material for: A Genome-Wide Association Study on Chronic HBV Infection and Its Clinical Progression in Male Han-Taiwanese
Source: PLoS One. 2014 Jun 18;9(6):e99724. doi: 10.1371/journal.pone.0099724 (PMC4062466; doi:10.1371/journal.pone.0099724)
Supplement: Table S4 — Association analysis of rs7756516 with clinical classifications in HBsAg positive carriers. (DOCX) [file pone.0099724.s007.docx]

**Table S4 Association analysis of rs7756516 with clinical classifications in HBsAg positive carriers**

| **Clinical**  **Group** | **Genotype Frequency for the group of interest** | | | **MAF** | **Genotype Frequency for the other two groups** | | | **MAF** | **Trend test**  **P-value** | **Testing for mode**  **of inheritance** | **P-value^a^** | **OR**  **(95% CI)^a^** |
| --- | --- | --- | --- | --- | --- | --- | --- | --- | --- | --- | --- | --- |
|  | **CC** | **CT** | **TT** |  | **CC** | **CT** | **TT** |  |  |  |  |  |
| PNALT | 0.015 | 0.170 | 0.815 | 0.100 | 0.004 | 0.132 | 0.865 | 0.073 | 0.0134 | Additive | 0.0249 | 0.74 (0.56-0.96) |
|  |  |  |  |  |  |  |  |  |  | Dominant | 0.0475 | 1.35 (1.00-1.80) |
|  |  |  |  |  |  |  |  |  |  | Recessive | 0.0851 | 2.63 (0.88-7.89) |
|  |  |  |  |  |  |  |  |  |  |  |  |  |
| CHB | 0.003 | 0.146 | 0.851 | 0.076 | 0.014 | 0.145 | 0.842 | 0.086 | 0.3259 | Additive | 0.1890 | 1.19 (0.92-1.55) |
|  |  |  |  |  |  |  |  |  |  | Dominant | 0.4048 | 1.13 (0.85-1.50) |
|  |  |  |  |  |  |  |  |  |  | Recessive | 0.0366 | 5.02 (1.11-22.83) |
|  |  |  |  |  |  |  |  |  |  |  |  |  |
| HCC | 0.011 | 0.111 | 0.877 | 0.067 | 0.008 | 0.156 | 0.837 | 0.086 | 0.1229 | Additive | 0.3365 | 1.18 (0.84-1.65) |
|  |  |  |  |  |  |  |  |  |  | Dominant | 0.2045 | 1.27 (0.88-1.83) |
|  |  |  |  |  |  |  |  |  |  | Recessive | 0.3822 | 1.72 (0.51-5.77) |

^a^ORs and CIs were calculated by setting the T allele as the reference with age adjustment.
