# Supplementary material for: A Genome-Wide Association Study on Chronic HBV Infection and Its Clinical Progression in Male Han-Taiwanese
Source: PLoS One. 2014 Jun 18;9(6):e99724. doi: 10.1371/journal.pone.0099724 (PMC4062466; doi:10.1371/journal.pone.0099724)
Supplement: Table S5 — Haplotype association of the 5 risk SNPs rs9276370, rs7756516, rs7453920 and rs9277535 with persistent HBV infection. (DOCX) [file pone.0099724.s008.docx]

**Table S5 Haplotype association of the 5 risk SNPs rs9276370, rs7756516, rs7453920 and rs9277535 with persistent HBV infection**

| **Haplotype** | **Estimated Frequency (%)** | | | **Logistic regression** | | **Logistic regression adjusted for age** | |
| --- | --- | --- | --- | --- | --- | --- | --- |
|  | **Case** | **Control** | **All** | **P-value** | **OR (95% CI)** | **P-value** | **OR (95% CI)** |
|  | (N=1623) | (N=1065) | (N=2688) |  |  |  |  |
| T-T-G-G | 69.93% | 58.26% | 65.31% | 1.95×10^-18^ | 1.67 (1.49-1.87) | 1.95×10^-18^ | 1.67 (1.49-1.87) |
| T-T-G-A | 21.66% | 26.95% | 23.75% | 8.53×10^-6^ | 0.75 (0.66-0.85) | 1.23×10^-5^ | 0.75 (0.66-0.86) |
| All other | 8.41% | 14.79% | 10.94% | 4.40×10^-13^ | 0.53 (0.45-0.63) | 1.87×10^-13^ | 0.52 (0.44-0.62) |
