# Supplementary material for: A Genome-Wide Association Study on Chronic HBV Infection and Its Clinical Progression in Male Han-Taiwanese
Source: PLoS One. 2014 Jun 18;9(6):e99724. doi: 10.1371/journal.pone.0099724 (PMC4062466; doi:10.1371/journal.pone.0099724)
Supplement: Table S6 — Haplotype association of 2 SNPs rs9276370 and rs7756516 with therapeutic response. (DOCX) [file pone.0099724.s009.docx]

**Table S6 Haplotype association of 2 SNPs rs9276370 and rs7756516 with therapeutic response**

| **Haplotype** | **Estimated Frequency (%)** | | | | **Logistic regression** | | **Logistic regression adjusted for age at treatment** | | |
| --- | --- | --- | --- | --- | --- | --- | --- | --- | --- |
|  | **Sustained** | **Non-sustained** | | **All** | **P-value** | **OR (95% CI)** | | **P-value** | **OR (95% CI)** |
|  | (N=57) | | (N=169) | (N=226) |  |  |  |  |  |
| T-T | 85.96% | | 92.60% | 90.93% | 0.0357 | 0.49 (0.25-0.95) | | 0.0262 | 0.46 (0.23-0.91) |
| G-C | 13.16% | | 5.92% | 7.74% | 0.0147 | 2.41 (1.19-4.88) | | 0.0132 | 2.49 (1.21-5.11) |
| All other | 0.88% | | 1.48% | 1.33% | 0.6319 | 0.59 (0.07-5.10) | | 0.7604 | 0.71 (0.08-6.31) |
